# Heart Disease

**Published:** 1884-06

**Authors:** 


### HEART DISEASE.

THERE are probably many among those who will read this article who believe themselves victims of heart disease. If a repetition of the statement we have repeatedly made, that examinations after death have shown that only one person in twenty of those who were supposed to have died of heart disease, had any disease whatever of that organ, will serve to allay the fears of a single *malade imaginaire* we shall feel amply rewarded for the trouble of writing it. When we consider that a large proportion of the adult population of this country suffers more or less from dyspepsia, and that that disease simulates heart trouble so closely in many cases as to require a careful examination by a physician to determine the question, we can not wonder at the supposed prevalence of a disease that is comparatively rare. But still there are cases of real heart disease, that with proper care cause no marked inconvenience or suffering, and where the patients live to old age or die of other maladies. Again, there are victims of the disease to whom death comes as a welcome relief.

Sometimes an heriditary tendency to the malady exists, as in consumption and other diseases ; but in the light of medical knowledge at the present time it could hardly be regarded as a fatality. Careful living usually overcomes such tendencies. A prominent man doing business in New York, but residing in the suburbs, having been delayed in his office a few minutes longer than usual, ran in order to be in time for his regular train : the following day he died of heart disease. Had he given the matter a thought, it probably would have occurred to him that the effect might very naturally follow the cause. A person past middle age, and unaccustomed to such vigorous exercise, can not hurry the circulation of the blood and the respiration with impunity.

Nothing is more dangerous than irregular and violent exercise for persons unaccustomed to it. Students and others of sedentary habits who exercise in gymnasiums, can hardly restrain themselves from efforts to excel ; and thus many bring injuries upon themselves that impair health and shorten life. As generally conducted, gymnasiums are responsible for a great amount of evil. If any special muscles are unduly developed, the development is at the expense of other muscles, and thus the equilibrium between the muscles of the body is deranged or destroyed. The heart is a muscle, and its development is a *disease* to which athletes and gymnasts are peculiarly liable.

We do not wish to be understood as saying that heart disease never comes from dyspepsia. Blood is made from the food taken into the stomach. If not properly digested, the quality of the blood is poor, the circulation is impaired, and the action of the heart is irregular. This would lead to disease, inevitably, did not the stomach rebel and positively enforce better treatment, and that is the reason so few dyspeptics suffer from actual disease of the heart ; although there may be great functional disturbance. It is a trite saying "that a gentleman is never in a hurry." He is supposed to do all things with dignity and deliberation. He never runs to catch a train, or jumps over the ferry-boat gates to get ashore first. He is never in haste to reach the dining room at the first tap of the bell, nor in a hurry to leave after he gets there. If he were generally adopted as a model there would be fewer cases of dyspepsia.

The great affairs of the world are conducted by men who are systematic and deliberate. Spasmodic energy upsets more than it builds up : this is a rule that applies to health as well as to affairs of business.

